# Natural Products as Mite Control Agents in Animals: A Review

**DOI:** 10.3390/molecules28196818

**Published:** 2023-09-27

**Authors:** Fei Liao, Changquan Han, Qingsheng Deng, Ziyao Zhou, Taotao Bao, Menghuai Zhong, Guangyao Tao, Renjun Li, Bo Han, Yanlong Qiao, Yanchun Hu

**Affiliations:** 1 Department of Animal Husbandry and Fisheries, Guizhou Vocational College of Agriculture, Qingzhen 551400, China; liaofeiyh@163.com (F.L.); hancqnzy@163.com (C.H.); nzydqs@163.com (Q.D.); 66zmh@163.com (M.Z.); tguangya0@icloud.com (G.T.); 11317035@zju.edu.cn (R.L.); hanbonzy@163.com (B.H.); 2 Key Laboratory of Animal Disease and Human Health of Sichuan Province, College of Veterinary Medicine, Sichuan Agricultural University, Chengdu 611130, China; zzhou@sicau.edu.cn; 3 Qiandongnan Center for Animal Disease Control and Prevention, Kaili 556000, China; baott-academic@outlook.com

**Keywords:** natural products, metabolites, acaricidal activity, acaricidal mechanism, animal mites

## Abstract

Mites have been a persistent infectious disease affecting both humans and animals since ancient times. In veterinary clinics, the primary approach for treating and managing mite infestations has long been the use of chemical acaricides. However, the widespread use of these chemicals has resulted in significant problems, including drug resistance, drug residues, and environmental pollution, limiting their effectiveness. To address these challenges, researchers have shifted their focus towards natural products that have shown promise both in the laboratory and real-world settings against mite infestations. Natural products have a wide variety of chemical structures and biological activities, including acaricidal properties. This article offers a comprehensive review of the acaricidal capabilities and mechanisms of action of natural products like plant extracts, natural compounds, algae, and microbial metabolites against common animal mites.

## 1. Introduction

There are many kinds of mites, with more than ten species related to human and animal health. Common mites include *Dermanyssus gallinae* (*D. gallinae*), *Posroptes cuniculi* (*P. cuniculi*), *Sarcoptes scabiei* (*S. scabiei*), *demodex*, etc. *D. gallinae* (chicken mite), a blood-sucking ectoparasite, infests poultry worldwide, and its infestation leads to reduced food intake, weakened immune response, and, in severe cases, death of chickens [1]. Moreover, *D. gallinae* infestation causes a decline in laying percentage and reduces egg weights [2]. *D. gallinae* infestation is a significant threat to poultry farms due to its ability to transmit various diseases and pathogens, including fowl spirochetosis, Newcastle disease virus, pullorum disease, and fowl plague. This causes substantial losses to the poultry industry in terms of both economic value and animal health [3]. According to an estimate, the European Union egg industry incurs an annual economic loss of about EUR 130 million related to control measures and production losses from *D. gallinae* infestations [4]. *P. cuniculi*, a parasitic mite, targets the external auditory canal of rabbits and feeds on serous exudate, secretions, and blood. Due to its high transmission rate and rapid spread, *P. cuniculi* poses a serious threat to the healthy development of the rabbit farming industry, necessitating effective control measures to mitigate detrimental effects [5]. *S. scabiei*, another pathogenic organism, causes a highly contagious skin disease in humans and numerous domestic and wild mammals [6]. This disease, known as sarcoptic mange, is characterized by irritation, inflammation, hyperkeratosis, alopecia, and excessive itching and often accompanies secondary infections [7]. Modeling studies have estimated that the global prevalence of scabies is between 100 and 200 million cases, with approximately 4.55 million new cases annually [8]. Furthermore, Sarcoptes infections significantly impact animal health and production, causing financial losses with serious animal welfare concerns. Infested animals experience reduced growth rates, decreased feed efficiency, and compromised immune function. The constant itching and irritation caused by mites lead to persistent discomfort and stress, affecting the overall well-being of the animals. These detrimental effects not only result in economic losses for the livestock industry, but also raise ethical concerns regarding animal treatment [9]. *Demodex* mites have co-evolved over millions of years as obligate commensal ectoparasites that inhabit the hair follicles and sebaceous glands of various mammalian species, including cats, dogs, cattle, horses, rabbits, and even humans [10]. *Demodex folliculorum* and *Demodex brevis* have been associated with ocular manifestations in humans [11], causing rosacea, blepharitis, keratitis, meibomian gland dysfunction, and inflammatory eyelid symptoms [12]. 

Mite control typically depends on the repeated use of conventional synthetic acaricides. However, their continuous use has led to the emergence of mite resistance, limiting their effectiveness. Additionally, the use of these chemicals has concerns about chemical residues in food and adverse environmental effects [13]. Therefore, there is a need for alternative strategies to manage mite infestations effectively. Lee et al. conducted a study in Korea and reported varying degrees of resistance to several acaricides in *D. gallinae* populations, including amitraz, milbemectin, clothianidin, thiamethoxam, and fenitrothion. This finding highlighted the problem of acaricide resistance, a significant challenge for the effective control of mite infestations in poultry farms, necessitating the exploration of alternative control measures [14]. Koc et al. investigated acaricide resistance mechanisms in *D. gallinae* populations in Turkey. They observed higher activity levels of detoxifying enzymes, specifically glutathione-S-transferases and carboxyl-cholinesterases, in these mite populations. Additionally, they discovered four target-site mutations associated with pyrethroid resistance, namely, M918T, T929I, F1534L, and F1538L, in domains II and III of the voltage-gated sodium channel. This finding uncovered the molecular mechanisms of acaricide resistance in Turkish *D. gallinae* populations, highlighting the need for alternative management strategies [15]. Schiavone et al. demonstrated that acaricide resistance in *D. gallinae* can be attributed to both target-site insensitivity and the overexpression of detoxification enzymes and other xenobiotic defense-related genes. These resistance mechanisms were found to be primarily constitutive, meaning that they were present in mites even in the absence of acaricide treatment and were not solely induced by exposure to acaricides. This suggested inherent genetic adaptations conferring resistance to acaricides in mites [16]. Ivermectin and permethrin are commonly used drugs to treat various parasitic infestations, including mite infections. However, their efficacy can be compromised due to the development of drug resistance in target mites [17]. Furthermore, these acaricides are ineffective against mite eggs and primarily target adult mites. The eggs may still hatch, leading to persistent infestation [18]. Feng et al. demonstrated that molting Sarcoptes mites have lower susceptibility to ivermectin compared to active mites [19]. The first report of in vitro resistance of *Psoroptes ovis* to ivermectin was documented in Argentina [20]. Romero et al. concluded that the residues of ivermectin used against Sarcoptes in lactating dairy goats were found in animal milk, raising concerns regarding the safety of milk intended for human consumption [21]. Acaricide resistance in mites can develop through mutations in the acaricide target site or upregulation of detoxification enzyme genes [22]. Therefore, there is a need to search for new bioactive agents with high efficiency, species selectivity, and good safety as an alternative to traditional chemical acaricides. Consequently, the use of natural products has gained increasing attention in the treatment of various ailments, in both humans and animals. Natural products have emerged as promising sources for small-molecule drug discovery in the field of acaricide development [23].

Natural products are components or metabolites derived from plants, microorganisms, insects, and marine organisms. They have characteristics of wide sources, diverse components, unique structures, low side effects, and reduced chances of drug resistance. Due to the complexity of natural metabolites, traditional bioassay-guided studies are considered laborious. However, the emergence of metabolomics research has attracted extensive interest for its ability to handle diverse metabolites [24]. High-resolution liquid chromatography-mass spectrometry (LC-HRMS) [25] and nuclear magnetic resonance (NMR) spectroscopy [26] are some of the commonly used analytical tools in metabolomics. Natural products have been reported for various biological activities, including anti-inflammatory [27], antioxidant [28], antibacterial [29], antiparasitic [30], antifungal [31], analgesic [32], anti-atherogenic [33], antidiabetic [34], and antiproliferative properties [35]. In recent years, several studies have reported that natural products also have anti-acaricidal activity with great application potential. 

This article provides a summary of the current research status on various types of natural products that possess acaricidal properties, including single-flavor Chinese herbal extracts, Chinese herbal monomers, and natural products derived from microbial and algae sources. This compilation aims to offer valuable insights and references for the development of novel acaricidal drugs.

## 2. Acaricidal Activity of Plant Extracts

Owing to adverse effects associated with the excessive use of chemical agents, such as drug resistance, residues, and environmental pollution, their usage has been restricted. As a result, there has been a growing interest in seeking alternative and natural acaricides derived from plants. Plant secondary metabolites, with toxic, repellent, attractant, and growth regulator properties, have been widely investigated for potential applications [36]. This section discusses various plant extracts and a brief detail is provided in Table 1.

### 2.1. Plant Extracts against D. gallinae 

**Figure 1:**
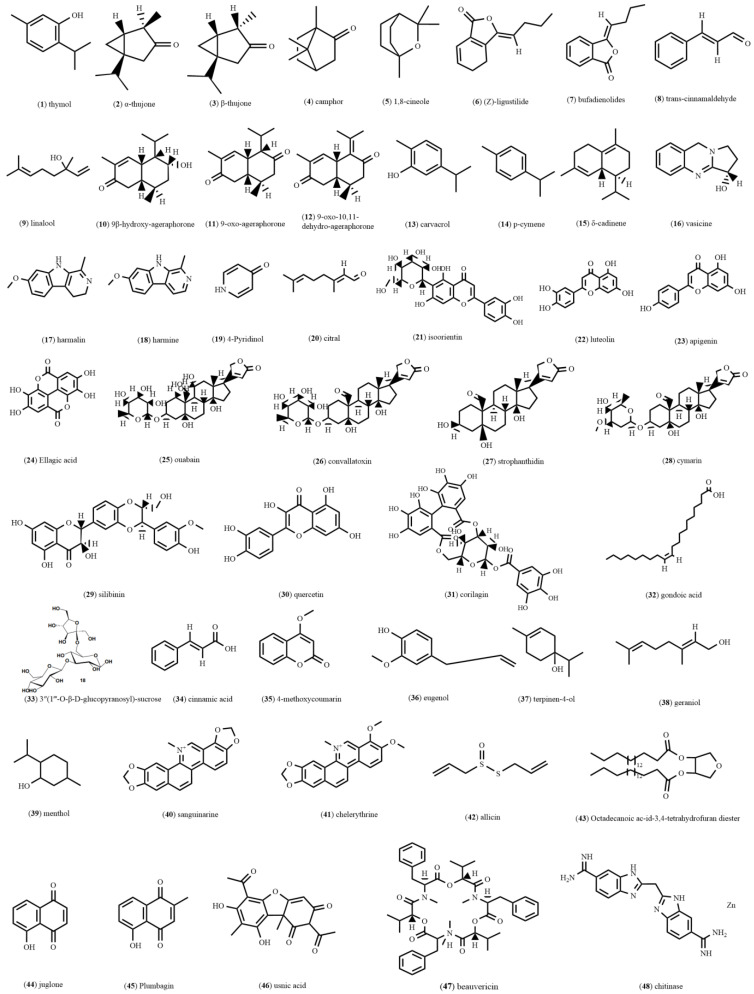
Structures of various natural compounds.

**Figure 2:**
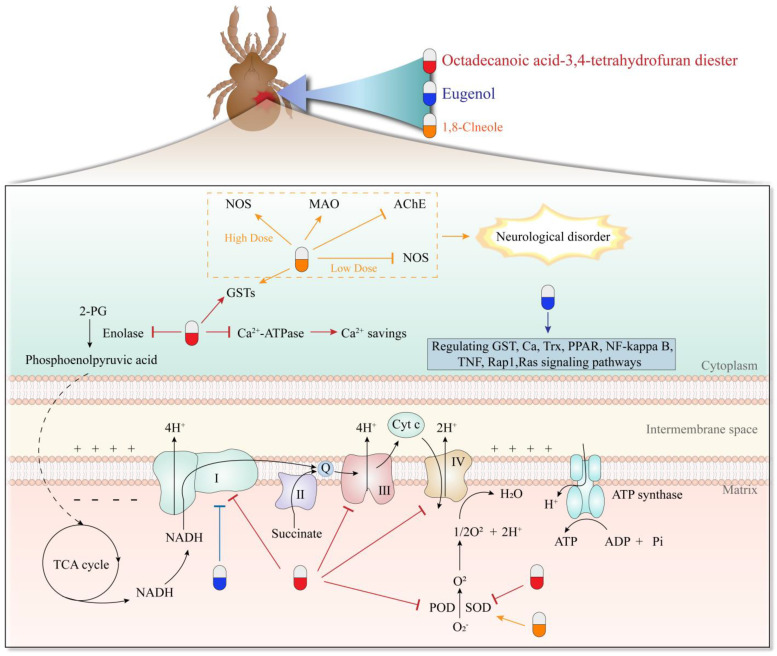
Acaricidal mechanism of some compounds.

Imani et al. demonstrated the potent acaricidal effects of essential oil (EO) and alcoholic extracts (AE) of Ajowan against *D. gallinae*. Thymol (Figure 1-(**1**)) was identified as the main constituent in both EO (42.26%) and AE (45.8%). Furthermore, they found that the spraying method was more effective than the contact method at 24 h post-treatment [13]. The toxicity of *Artemisia sieberi* (*A. sieberi*) EO against *D. gallinae* was evaluated through contact and fumigant assays on adult mites. The *A. sieberi* EO contains α-thujone (Figure 1-(**2**); 31.5%), β-thujone (Figure 1-(**3**); 11.92%), camphor (Figure 1-(**4**); 12.3%), and 1,8-cineole (Figure 1-(**5**); 10.09%). The contact toxicity assay on adult mites demonstrated a median lethal concentration (LC50) value of 15.85 μg/cm^3^ for the EO [37]. Kim et al. [1] evaluated the efficacy of methanolic extract (ME) from *Cnidium officinale* (*C. officinale*) against *D. gallinae* adults. At 4000 ppm (parts per million), ME led to a 100% mortality rate after 48 h of treatment, and it mainly contains (Z)-ligustilide (Figure 1-(**6**)). Notably, the extract of *C. officinale* exhibited both fumigant and contact activities, whereas (Z)-ligustilide demonstrated only the fumigant activity. Pares et al. were the first to report the acaricidal activity of *Xylopia emarginata* (*X. emarginata*) against *D. gallinae*, with an LC50 value of 331.769 μg/cm^2^. Moreover, metabolomic profile analysis of *X. emarginata* revealed the presence of a diverse range of compounds, including amides, alkaloids, phenolics, and terpenoids [38]. The extract of *Drimia maritima* (*D. maritima*) exhibited remarkable acaricidal activity against *D. gallinae*, achieving a 100% mortality rate at 100 mg/mL after 24 h of treatment. This high efficacy of *D. maritima* extract was attributed to bufadienolides (Figure 1-(**7**)) [39]. The EO extracts from *Clausena anisata*, *Litchi chinensis*, *Lippia alba*, *Heracleum sphondylium*, *Pimpinella anisum*, *Crithmum maritimum*, and *Syzygium aromaticum* were tested for their contact toxicity against *D. gallinae*, a deleterious ectoparasite of aviary systems. *Litchi chinensis* and *Syzygium aromaticum* were highlighted as two promising biopesticide sources for developing effective control strategies against *D. gallinae* infestations [40]. Alimi et al. evaluated the in vitro acaricidal activity of EO and crude extracts from *Laurus nobilis* and found them effective against *D. gallinae* in a contact toxicity test. *Laurus nobilis* extract mainly contained 1.8-cineole (46.56%) [41]. Furthermore, EO from *Cinnamomum cassia* (*C. cassia*) (LC50, 25.43 ± 1.0423 μg/cm^3^) and *Cinnamomum camphora var. linalooliferum* (*C. camphora var. linalooliferum*) (LC50, 39.84 ± 1.9635 μg/cm^3^) were found to be most active in the fumigant bioassay, with mortality rates of 96% and 61%, respectively. Gas chromatography-mass spectrometry (GC-MS) analysis revealed that the major constituents of EO from *C. cassia* and *C. camphora var. linalooliferum* were trans-cinnamaldehyde (Figure 1-(**8**)) and linalool (Figure 1-(**9**)), respectively [42].

### 2.2. Plant Extracts against P. cuniculi and S. scabiei

Many studies have demonstrated the high toxicity of certain plant extracts towards *P. cuniculi* and *S. scabiei*, including those from *Syzygium aromaticum*, *Eupatorium adenophorum* (*E. adenophorum*), *Azadirachta indica*, and *Adonis coerulea*. *E. adenophorum* has emerged as a significant weed in various agricultural settings, plantations, natural habitats, and forests across multiple regions globally. It is particularly considered the most impactful invasive species in China. Notably, AE derived from *E. adenophorum* exhibited potent toxicity against mites, effectively eliminating all *S. scabiei* at 0.5 and 1.0 g/mL (*w*/*v*). Furthermore, at 1 g/mL, the extract demonstrated complete eradication of *P. cuniculi* within a short span of 4 h, and the insecticidal effect of its 9β-hydroxy-ageraphorone compound (Figure 1-(**10**)) was better than the insecticidal effect of fenvalerate and 9-oxo-ageraphorone compounds at 0.5% (Figure 1-(**11**)). Moreover, 9-oxo-10,11-dehydro-ageraphorone (Figure 1-(**12**)) exhibited higher insecticidal effects than 9β-hydroxy-ageraphorone [43]. *Origanum vulgare* (Oregano, *Labiateae* family) oil showed remarkable acaricidal effects against *P. cuniculi* in a dose- and time-dependent manner, and it majorly contains carvacrol (Figure 1-(**13**)), thymol, and p-cymene (Figure 1-(**14**)). In vitro experiments demonstrated that at 0.05% and 0.02% (*v*/*v*), oregano oil resulted in complete eradication of *P. cuniculi* within 1 h and 6 h, respectively. Furthermore, a clinical evaluation was conducted using naturally infected rabbits to assess the efficacy of oregano oil. At 1% and 5%, oregano oil completely eliminated *P. cuniculi* infestation in rabbits, improving animal mental and physical conditions by the end of the study on the 20th day [44]. The EO extracts obtained from the shoots and leaves of *Rhododendron nivale* Hook.f. (*R. nivale*) exhibited potent in vitro acaricidal activity against adult *P. cuniculi*, with an LT50 value of up to 4.17 mg/mL. Following the EO treatment, *P. cuniculi*-infected rabbits showed a complete absence of scabs or secretions in the ear canal by the 20th day of treatment. The EO of *R. nivale* mainly contains δ-cadinene (Figure 1-(**15**)) and displayed pronounced acaricidal activity against *P. cuniculi* in vitro [45]. *Peganum harmala* L., a perennial herbaceous plant, grows in semi-arid conditions, steppe areas, and sandy soils. Shang et al. evaluated the acaricidal activity of microwave-assisted extract (MAE) from *Peganum harmala* L. against *P. cuniculi* in vitro. The LT50 value of 100 mg/mL MAE extract against *P. cuniculi* was 17.322 h. The main active ingredients identified in the MAE extract were vasicine (Figure 1-(**16**)), harmaline (Figure 1-(**17**)), and harmine (Figure 1-(**18**)) [46]. 

Gu et al. reported the acaricidal activity of *Ailanthus altissima* (*A. altissima*) bark AE against *P. cuniculi* and *S. scabiei*. The LT50 values of the *A. altissima* bark AE against *S. scabiei* were 0.60, 0.78, and 1.48 h at 1, 0.5, and 0.25 g/mL (*w*/*v*), respectively. For *P. cuniculi*, the median lethal time (LT50) values were 0.74, 1.29, and 3.33 h at the same concentrations [47]. Andriantsoanirina et al. evaluated the acaricidal activity of 31 EOs from different plants against *S. scabiei*. *Cinnamomum zeylanicum* and *Ocimum sanctum* EOs were found to be most active at 10 to 0.1% (*v*/*v*) [48]. Fang et al. assessed the potential efficacy of ten EO against *S. scabiei*. Among the tested oils, 1% (*v*/*v*) clove oil and palmarosa oil demonstrated high effectiveness, killing all mites within 20 and 50 min, respectively. The order of efficacy for the tested oils was: clove > palmarosa > geranium > tea tree > lavender > manuka > bitter orange > eucalyptus > Japanese cedar. Notably, cade oil exhibited no activity against *S. scabiei* [49]. The AE of *Ligularia virgaurea* demonstrated potent acaricidal activity against *S. scabiei*. Its LC50 values against mites at different time intervals were as follows: 1.388 g/mL at 1 h, 0.624 g/mL at 2 h, 0.310 g/mL at 4 h, and 0.213 g/mL at 6 h [50]. An in vitro study found a significant acaricidal activity of *Elsholtzia densa* (*E. densa*) Benth oil: its LC50 values against *S. scabiei* at different time intervals were as follows: 7.678 mg/mL at 1 h, 4.623 mg/mL at 2 h, 2.543 mg/mL at 4 h, 1.502 mg/mL at 8 h, 1.298 mg/mL at 16 h, and 0.981 mg/mL at 24 h. GC-MS analysis of the Benth oil revealed that it primarily contained 4-pyridinol (Figure 1-(**19**); 28.16%) and thymol (26.58%) [51]. Lemongrass oil exhibited potent acaricidal activity against *Sarcoptes* mites. At 10% and 5% (*v*/*v*), it killed all mites within 10 and 25 min, respectively. Its LC50 values were 1.37%, 1.08%, 0.91%, 0.64%, and 0.48% at 1, 3, 6, 12, and 24 h, respectively. Moreover, lemongrass oil significantly reduced the hatching rate of *Sarcoptes* eggs at various concentrations (10%, 5%, 1%, 0.5%, and 0.1% *v*/*v*). Mass spectrometry analysis confirmed that the main component in lemongrass oil is citral (Figure 1-(**20**)) [52]. In a comparative study, three concentrations (5%, 10%, and 25% *w*/*v*) of aqueous neem fruit extract were assessed against commercial acaricide called 12.5% amitraz-based Triatix spray (used as the positive control) on pigs. The study found that the topical application of the 25% aqueous neem fruit extract demonstrated a higher efficacy against mites than a commercial acaricide [53]. *Adonis coerulea Maxim.* (*A. coerulea*) exhibited acaricidal activity in both in vitro and in vivo. It inhibited acetylcholinesterase (AchE) and Na^+^-K^+^-ATPase enzymes activities, and mainly contained isoorientin (Figure 1-(**21**)), luteolin (Figure 1-(**22**)), apigenin (Figure 1-(**23**)), ellagic aci (Figure 1-(**24**)), ouabain (Figure 1-(**25**)), convallatoxin (Figure 1-(**26**)), strophanthidin (Figure 1-(**27**)), and cymarin (Figure 1-(**28**)) [54,55,56]. Dai et al. used LC-MS/MS and molecular docking to study the mechanism of ME from *A. coerulea* on AchE, which mainly contained silibinin (Figure 1-(**29**)), quercetin (Figure 1-(**30**)), and corilagin (Figure 1-(**31**)). They found that silibinin, quercetin, and corilagin could inhibit AchE activity at the cellular level, with IC50 values of 40.11, 46.15, and 50.98 μg/mL, respectively [57]. The coconut seed extract was shown to have acaricidal activity against *S. scabies* in vitro and in vivo. In vitro, the mortality rate of mites reached 99% after 1 day, and the mRNA gene expression results showed that IL-6, IL-1β, IL-10, MMP-9, VEGF, and MCP-1 were inhibited, while I-CAM-1, KGF, and TIMP-1 were upregulated. The results of molecular docking analysis showed that the main substances in coconut seed extract that killed mites were gondoic acid (Figure 1-(**32**)) and 3″(1‴-O-β-d-glucopyranosyl)-sucrose (Figure 1-(**33**)) [58].

**Table 1:** A summary of the acaricidal activity of plant extracts.

Extracts	Main Components	Mite	Acaricidal Dose	Mechanism of Action	Reference
EO and AE of Ajowan	Thymol	*D. gallinae*	At 24 h post-treatment, EO and AE both exceeded 90% mortality at 50 μg/cm^2^ and 150 μg/cm^2^, respectively	/	[13]
EO of *A. sieberi*	α-thujone (31.5%), β-thujone (11.92%), camphor (12.3%), 1,8-cineole (10.09%)	*D. gallinae*	LC50 15.85 μg/cm^3^	/	[37]
ME of *C. officinale*	(Z)-ligustilide	*D. gallinae*	After 48 h of treatment, 100% mortality at 4000 ppm	/	[1]
ME of *X. emarginata*	Amides, alkaloids, phenolic, and terpenoids	*D. gallinae*	LC50 331.769 μg/cm^2^	/	[38]
Acetonic extract of *D. maritima* bulbs	Bufadienolides	*D. gallinae*	At 100 mg/mL, the mortality was 100% after 24 h of exposure	/	[39]
EO of *Syzygium aromaticum* and *Litchi chinensis*	/	*D. gallinae*	LC50 8.9–24.7 μg/mL	/	[40]
*Laurus nobilis* essential oil	1.8-cineole	*D. gallinae*	After 12 h of treatment, 100% mortality at 320 mg/mL	/	[41]
EO of *C. cassia*	Trans-cinnamaldehyde	*D. gallinae*	LC50 25.43 ± 1.0423 μg/cm^3^	/	[42]
EO of *C. camphora var. linalooliferum*	Linalool	*D. gallinae*	LC50 39.84 ± 1.9635 μg/cm^3^	/	[42]
*E. adenophorum*	9-oxo-ageraphorone, 9-oxo-10,11-dehydro-ageraphorone, and 9β-hydroxy-ageraphorone	*P. cuniculi* and *S. scabiei*	0.5%	/	[43]
Oregano oil	Carvacrol, thymol, and p-cymene	*P. cuniculi*	0.05% and 0.02% (*v*/*v*) killed all mites within 1 and 6 h, respectively	/	[44]
EO of *R. nivale*	δ-cadinene	*P. cuniculi*	LT50 values of (33.33–4.17 mg/mL) of the EO ranged from 1.476 to 25.900 h	/	[45]
MAE extract of *Peganum harmala L.*	Vasicine, harmaline, and harmine	*P. cuniculi*	LT50 value of 100 mg/mL MAE extract against *P. cuniculi* was 17.322 h	/	[46]
AE of *Ailanthus altissima bark*	/	*P. cuniculi*	LT50 values at 1, 0.5, and 0.25 g/mL were 0.74, 1.29, and 3.33 h, respectively	/	[47]
AE of *Ailanthus altissima bark*	/	*S. scabiei*	LT50 values at 1, 0.5, and 0.25 g/mL were 0.60, 0.78, and 1.48 h, respectively	/	[47]
*Cinnamomum zeylanicum* and *Ocimum sanctum* EOs	/	*S. scabiei*	Most active at 10–0.1%	/	[48]
Clove oil and palmarosa oil	/	*S. scabiei*	1% clove and palmarosa oil killed all mites within 20 and 50 min, respectively	/	[49]
AE of *Ligularia virgaurea*	/	*S. scabiei*	LC50 values were 1.388, 0.624, 0.310, and 0.213 g/mL at 1, 2, 4, and 6 h, respectively	/	[50]
EO of *Elsholtzia densa* (*E. densa*) Benth	4-Pyridinol (28.16%) and thymol (26.58%)	*S. scabiei*	LC 50 values were 7.678–0.981 mg/mL at 1–24 h	/	[51]
Lemongrass oil	Citral	*S. scabiei* and *S. scabiei* eggs	*S. scabiei*: LC50 1.37%, 1.08%, 0.91%, 0.64%, and 0.48% at 1, 3, 6, 12, and 24 h, respectively; for eggs: 10%, 5%, 1%, 0.5%, and 0.1%, respectively	Decreases the hatching rate	[52]
Aqueous neem fruit extracts	/	*S. scabiei*	25%	/	[53]
ME of *Adonis coerulea* Maxim	Isoorientin, luteolin, and apigenin	*P. cuniculi*	/	Inhibits AchE and Na^+^-K^+^-ATPase activities	[54]
ME of *Adonis coerulea* Maxim	Ellagic acid, ouabain, convallatoxin, strophanthidin, and cymarin	*P. cuniculi*	At 100 mg/mL, the mortality was 55.00% after 24 h	Inhibits Na^+^-K^+^-ATPase	[56]
ME of *Adonis coerulea* Maxim	Silibinin, quercetin, and corilagin	*P. cuniculi*	Inhibit AchE activity with IC50 values of 40.11, 46.15, and 50.98 μg/mL, respectively	Inhibits AchE	[57]
Coconut seed extract	Gondoic acid and 3″(1‴-O-β-d-glucopyranosyl)-sucrose	*S. scabiei*	/	Inhibits IL-1β, IL-6, IL-10, MMP-9, VEGF, and MCP-1; upregulates I-CAM-1, KGF, and TIMP-1	[58]

Note: EO: essential oil; AE: alcoholic extracts; ME: methanolic extract; MAE: microwave-assisted extract; slash (/) denotes an unknown mechanism.

The wide cultivation of the above-discussed natural products and their extensive use in foodstuffs and cosmetics as flavors and fragrances suggest that they could serve as a cost-effective and easily accessible eco-friendly alternative to the currently used pesticides in poultry farms. However, it is important to note that the extract composition is complex, and further studies are needed to identify and understand the specific medicinal substances present in these extracts. Future studies would provide a more comprehensive understanding of their potential as pesticides and facilitate their sustainable utilization in poultry farms.

## 3. Acaricidal Activity of Natural Compounds

Various compounds from natural sources with acaricidal activity are discussed in this section and a brief description of the chemicals is listed in Table 2. The acaricidal mechanisms of the compounds are listed in Figure 2.

### 3.1. Phenylpropanoids

Among the phytochemicals found in EOs, cinnamaldehyde, an α,β-unsaturated aldehyde, has been noted for its remarkable antimicrobial properties and its ability to enhance the effectiveness of antibiotics. Cinnamic acid (Figure 1-(**34**)) and its esters, which are widely distributed in plants, have garnered significant attention due to their diverse pharmacological activities. Trans-cinnamaldehyde and ethyl cinnamate, analogs of cinnamic acid, have also been reported for acaricidal activity. Cinnamaldehyde is particularly abundant in EOs from *Cinnamomum* species. Notably, it is widely used as a food additive, and its antimicrobial activity makes it a valuable component for potential applications in both the medical and food industries [59]. In vitro studies were performed to assess the acaricidal activity of trans-cinnamaldehyde against *P. cuniculi*, and the results showed that trans-cinnamaldehyde up to 8 μg/mL exhibited significant mites mortality rate (*p* < 0.01) [60]. Ethyl cinnamate derivatives have been identified as promising and highly efficient acaricides against *P. cuniculi*. The structure–activity relationship (SAR) analysis revealed that the presence of o-NO^2^ or m-NO^2^ on their benzene ring significantly enhanced their activity. On the other hand, the introduction of a hydroxy, methoxy, acetoxy, methylenedioxy, bromo, or chloro group reduced the activity [61]. Chen et al. [62] synthesized cinnamic acid derivatives and isoaromatic ring analogs to evaluate their in vitro acaricidal activities against *P. cuniculi*. Among them, eight compounds exhibited higher activity. Structure–activity relationship (SAR) analysis revealed the crucial role of the carbonyl group in the activity. Also, the type and chain length of the alkoxy group in the ester moiety, as well as the steric hindrance near the ester group, significantly influenced the activity. Ester derivatives demonstrated greater activity compared to thiol esters, amides, ketones, or acids. Substituting the phenyl group of cinnamic esters with a-pyridyl or a-furanyl groups led to a significant increase in activity. Shang et al. investigated the anti-pruritus activity of 18 coumarins and found that among the coumarins, 4-methoxy-coumarin (Figure 1-(**35**)) exhibited the highest anti-mite activity, with an LC50 value of 34.00 μg/mL. Importantly, 4-methoxy-coumarin demonstrated minimal to no toxicity towards normal human hepatocytes and keratinocytes, with an LC50 value of greater than 100 μg/mL. This finding suggested that 4-methoxy-coumarin holds great potential for further research and development, particularly in the context of managing pruritus [63]. Eugenol (Figure 1-(**36**)), a naturally occurring phenolic monoterpenoid, has bioactive properties and belongs to the phenylpropanoids class of natural products. It is commonly found in various aromatic herbal plants, including clove, tulsi, cinnamon, nutmeg, and pepper. However, it is primarily isolated from the clove plant (*Syzygium aromaticum*). Eugenol has a broad range of applications in various industries such as pharmaceuticals, food, flavors, cosmetics, agriculture, and many others. Eugenol is well-known for its diverse pharmacological properties, including antimicrobial, anticancer, antioxidant, anti-inflammatory, and analgesic effects [64]. Ma et al. showed that eugenol can completely eradicate *P. cuniculi* mites at 4 mg/mL for 8 h. The median lethal dose (LD50) of eugenol ranged from 1.564 ± 0.023 to 1.039 ± 0.009 mg/mL at 1 to 24 h after treatment. The study suggested that several signaling pathways, including PPAR (peroxisome proliferator-activated receptor), NF-kappa B, TNF (tumor necrosis factor), Rap1, and Ras pathways, might play significant roles in mite killing by eugenol [5]. For instance, eugenol inhibited complex I activity of the mitochondrial respiratory chain in the oxidative phosphorylation pathway by binding to NADH dehydrogenase chain 2, causing the death of mites [55]. The mite’s inhibitory activity of five compounds, terpine-4-ol (Figure 1-(**37**)), citral, linalool, eugenol, and geranyl (Figure 1-(**38**)) on eggs and eggs of naturally infected rabbits, was determined. The results showed that the median effect concentration (EC50) of eugenol, geranyl, citral, terpine-4-ol, and linalool were 0.65%, 0.66%, 0.85%, 1.47%, and 2.87%, respectively [65].

### 3.2. Terpenoids

Thymol, a monoterpene found in many natural plant EOs, has been discovered to possess strong toxicity against the scabies mites. In a study, the LC50 value of thymol against scabies mites was determined to be 3.829 mg/mL within a 4 h exposure. The mechanism of thymol’s acaricidal activity involves interference with the energy metabolism and nerve conduction of the mites [66]. 1,8-Cineole, a monoterpene, is found in many EOs and can change the activity of superoxide dismutase (SOD), nitric oxide synthase (NOS), and GSTs in the nervous system of *S. scabies* [67]. The acaricidal activity of 9-oxo-10,11-dehydroageraphorone (euptox A), a cadinane sesquiterpene derived from *E. adenophorum*, was tested against *S. scabiei* and *P. cuniculi* both in vitro and in vivo. In vitro studies revealed that euptox A killed all *S. scabiei* mites at 3–4 mg/mL and demonstrated complete lethality against *P. cuniculi* at 4 mg/mL within 4 h of the treatment [68]. In vivo, euptox A exhibited superior clinical acaricidal efficacy against *P. cuniculi* at 2 mg/mL. Concisely, euptox A has strong potential as an acaricidal agent against both *S. scabiei* and *P. cuniculi* [69]. Tabari et al. evaluated the efficacy of different combinations of terpenes, such as carvacrol, thymol, and menthol (Figure 1-(**39**)) against *D. gallinae* and found that the combination of all three was most effective compared to other combinations. This particular combination successfully killed 100% of the mites at 0.5 μg/mL. Additionally, the terpene-based combination exhibited promising miticidal activity in field conditions resulting in residue-free eggs, indicating its potential in environmentally friendly pest management practices [70]. Li et al. evaluated the activity of six commonly found terpenes in EOs (carvacrol, eugenol, geraniol, citral, terpine-4-ol, and linalool) against *S. scabies* adults and eggs. The EC50 values of carvacrol, eugenol, geranyl, citral, terpine-4-ol, and linalool were 0.5, 0.9, 2.0, 4.8, 5.1, and 9.8% (*w*/*v*), respectively. These terpenoids may act by penetrating the aerogen membrane on the egg surface. Importantly, carvacrol, eugenol, and geraniol showed significant ovicidal activity [71]. The LC50 values of carvacrol, eugenol, and geraniol at 30 min were 0.24%, 0.79%, and 0.91% (*w*/*v*), respectively [9].

### 3.3. Alkaloid Compounds

Alkaloids are important nitrogen-containing natural organic compounds that are widely distributed in plants. Shang et al. evaluated the acaricidal activity of three bioactive alkaloids from *Peganum harmala* L., namely, vasicine, harmaline, and harmine, against *P. cuniculi* in vitro. The LT50 values of vasicine, harmaline, and harmine against *P. cuniculi* at 2.5 mg/mL were 9.791, 10.095, and 9.273 h, respectively [46]. Sanguinarine (Figure 1-(**40**)) and chelerythrine (Figure 1-(**41**)) are two quaternary benzo[c]phenanthridine alkaloids that are widely present in several plant species of the Fumariaceae, Papaveraceae, and Rutaceae families. Miao et al. synthesized derivatives by modifying the C=N^+^ bonds of sanguinarine and chelerythrine, and then evaluated their in vitro acaricidal activity against *P. cuniculi*. A derivative, named 6-alkoxy dihydrosanguinarines, exhibited strong acaricidal activity against *P. cuniculi* at 5.0 mg/mL, which was comparable to the commercial acaricide ivermectin. The modification at the C=N^+^ double bond in sanguinarine and chelerythrine determined the acaricidal properties of the derivatives, and quaternary benzo[c] phenanthridine alkaloids showed promise for the development of new isoquinoline acaricidal agents [72].

### 3.4. Other Active Substances

A study investigated the efficacy of the ivermectin-allicin (Figure 1-(**42**)) combination against *D. gallinae*. Specifically, 0.5 mg/mL each of ivermectin and allicin completely eliminated *D. gallinae* within 5 days of treatment. The most effective combination was 0.25 mg/mL ivermectin with 1.00 mg/mL allicin [73]. Kang et al. evaluated the efficacy of ivermectin and allicin combinations against *D. gallinae* in vivo. A solution containing 0.25 mg/mL ivermectin and 1 mg/mL allicin (IA) was sprayed on hens housed in isolators that were infested with *D. gallinae*. The researchers found that IA exhibited insecticidal rates of 98.7%, 98.4%, 99.4%, and 99.9% at 7, 14, 21, and 28 days of treatment, respectively. Importantly, no clinical symptoms related to IA compounds or residues of ivermectin were observed in the treated hens [74]. Octadecanoic acid-3,4-tetrahydrofuran diester (Figure 1-(**43**)) is a newly identified compound from neem oil. Its acaricidal activity against *S. scabiei* is significantly higher compared to pyrethrins and abamectin. Its LC50 value (0.1 mg/mL) is approximately 1/33 of the neem oil. Mechanistically, this compound significantly changes the activity of mite enzymes, such as superoxide dismutase, peroxidase, Ca^2+^-ATPase, glutathione-s-transferases, and peroxidase. This suggests that octadecanoic acid-3,4-tetrahydrofuran diester may regulate the energy metabolism in mites [75,76]. Song et al. [77] conducted transcriptome and proteomics studies to investigate the acaricidal mechanism of octadecanoic acid-3,4-tetrahydrofuran diester. They identified several target proteins of the compound, including NADH dehydrogenase, ubiquinol-cytochrome c reductase, cytochrome c oxidase, ATP synthase, enolase, and superoxide dismutase. These findings suggest that the acaricidal mechanism of octadecanoic acid-3,4-tetrahydrofuran diester involves the interference with energy metabolism, particularly the oxidative phosphorylation pathway. Li et al. [78] modified the structure of octadecanoic acid-3,4-tetrahydrofuran diester to enhance its acaricidal activity, specifically by introducing benzyloxy substitution at the 2-position of the furan ring and forming a benzoate at the 3,4-position of the furan ring (benzoic acid-2-benzyloxy-3,4-tetrahydrofuran diester). Transcriptome sequencing analysis revealed that the acaricidal mechanism of this derivative involves interfering with energy metabolism in *S. scabiei*, particularly the citric acid cycle, oxidative phosphorylation pathway, and fatty acid metabolism. This finding was further confirmed through the activity detection of mitochondrial complexes. Naphthoquinone is an important secondary metabolite in plants with diverse biological activities. Shang et al. investigated the acaricidal activity of naphthoquinones against *P. cuniculi* both in vitro and in vivo. They found that juglone (Figure 1-(**44**)) and plumbagin (Figure 1-(**45**)) exhibited the strongest acaricidal activities against *P. cuniculi*, with LC50 values of 20.53 ppm and 17.96 ppm, respectively at 24 h. In the in vivo experiments, after three treatments, both juglone and plumbagin completely cured naturally infested rabbits within 15 days. Importantly, no skin irritation was observed in any of the treated rabbits, indicating the safety of these chemicals. Furthermore, the researchers discovered that juglone and plumbagin significantly inhibited the activity of AchE and GST, highlighting their mechanism [79].

**Table 2:** A summary of the acaricidal activity of natural compounds.

Compound Name	Classification	Mite	Acaricidal Dose	Mechanism of Action	Reference
Trans-cinnamaldehyde	Phenylpropanoids	*P. cuniculi*	Up to 8 μg/mL	/	[60]
4-methoxycoumarin	Phenylpropanoids	*P. cuniculi*	LC50 34.00 μg/mL	/	[63]
Eugenol	Phenylpropanoids	*P. cuniculi*	LD50 values at 1–24 h after treatment were 1.564 ± 0.023 to 1.039 ± 0.009 mgmL^−1^	Through PPAR, NF-kappa B, TNF, Rap1, and Ras signaling pathways	[5]
Eugenol	Phenylpropanoids	*P. cuniculi*	The inhibition rates were 37.89% for 50 μg/mL and 60.26% for 100 μg/mL, respectively	Inhibits complex I activity of the mitochondrial respiratory chain in the oxidative phosphorylation pathway	[55]
Eugenol, geraniol, citral, terpinen-4-ol, and linalool	Phenylpropanoids	*P. cuniculi* eggs	EC50 of egg hatching was 0.65–2.87%	/	[65]
Thymol	Monoterpene	*S. scabiei*	LC50 values were 3.829 mg/mL for *S. scabiei* in 4 h	Interference with the energy metabolism and nerve conduction of the mites	[66]
1,8-Cineole	Monoterpene	*S. scabiei*	LC50 and LT50 values were 2.77 mg/mL and 3.606 h, respectively	Changes activity of SOD, NOS, and GSTs activity in the nervous system	[67]
Euptox A	Sesquiterpene	*P. cuniculi* and *S. scabiei*	LC50 values were 1.068 mg/mL for *S. scabiei* and 0.902 mg/mL for *P. cuniculi* in 2 h	/	[68,69]
Combinations of carvacml-thymol-menthol	Terpenes	*D. gallinae*	100% killing at 0.5 μg/mL	/	[70]
Carvacrol, eugenol, geraniol	Terpenes	*S. scabiei* eggs	EC50 values were 0.5, 0.9, and 2.0% for carvacrol, eugenol, and geraniol, respectively	Penetrates through aeropyles on the egg surface	[71]
Carvacrol, eugenol, geraniol	Terpenes	*S. scabiei*	LC50 values at 30 min were 0.24, 0.79, and 0.91%, respectively	/	[9]
Vasicine, harmaline, harmine	Alkaloid	*P. cuniculi*	LT50 values at 2.5 mg/mL against *P. cuniculi* were 9.791, 10.095, and 9.273 h, respectively	/	[46]
Combinations of ivermectin-allicin	Organosulfur compound	*D. gallinae*	0.25 mg/mL ivermectin + 1.00 mg/mL allicin	/	[74]
Octadecanoic acid-3,4-tetrahydrofuran diester	Esters	*S. scabiei*	LC50 0.082 mg/mL at 24 h	Suppresses SOD, POD, and Ca(^2+^)-ATPase and activates GSTs	[75,76]
Octadecanoic acid-3,4-tetrahydrofuran diester	Esters	*S. scabiei*	/	Interferes with energy metabolism, especially oxidative phosphorylation pathway	[77]
Juglone	Naphthoquinones	*P. cuniculi*	LC50 20.53 ppm at 24 h	Inhibits AchE and GST activity	[79]
Plumbagin	Naphthoquinones	*P. cuniculi*	LC50 17.96 ppm at 24 h	Inhibits AchE and GST activity	[79]

Note: Slash (/) denotes an unknown mechanism.

## 4. Acaricidal Activity of Lichens and Algae

Algae contain natural active substances that are absent in terrestrial plants. In recent years, numerous studies have reported various active substances derived from algae with a wide range of biological activities, including antioxidant, anti-inflammatory, antimicrobial, antiviral, anticancer, neuroprotective activities, etc. The bioactive compounds found in algae, such as polysaccharides, polyphenols, pigments, and fatty acids, exert these biological effects [80]. Lectins are widely distributed in nature and can be found in plants, animals, and microorganisms. Among microorganisms, algae, particularly red algae, are known to be a potent source of lectins with unique properties [81]. The lectin derived from *Gracilaria ornata*, a type of red algae, has been found to possess acaricidal activity. Exposure of this lectin to female cattle ticks (*Boophilus microplus*) significantly reduced tick weight after the oviposition period, egg mass weight, hatching period, and mean larvae survival time [82]. A study reported the inhibitory effects of water-soluble *Moringa oleifera* (*M. oleifera*) lectin on egg hatching and larval development of gastrointestinal nematodes in goats, which function by interfering with the activity of parasites proteases and making potential interactions with intestinal receptors and larval cuticles [83]. Medeiros et al. demonstrated that proteins from *M. oleifera*, specifically water-soluble *M. oleifera* lectin and coagulant *M. oleifera* lectin, have inhibitory effects on infective larvae and adult male and female worms of *Haemonchus contortus*, a hematophagous parasite in ruminant animals. These lectins induce morphological changes in the worms and increase proteolytic activity [84].

Lichens are a rich source of natural products, including a wide variety of unique polyketides and polyphenols [85]. Usnic acid (Figure 1-(**46**)), a major active compound found in lichens, was first isolated in 1884 and is considered one of the best-studied lichen metabolites. Usnic acid has a wide range of biological activities, including anti-inflammatory, antibacterial, antiviral, immunostimulating, antifungal, and antiparasitic properties [86]. Shang et al. conducted an in vitro investigation on the acaricidal activity of usnic acid against *P. cuniculi*. They found that at 250, 125, and 62.5 mg/mL, usnic acid exhibited mite mortality rates of 91.67%, 85.00%, and 55.00%, respectively after a 24 h treatment period: the LT50 values of usnic acid were 4.208, 8.249, and 16.950 h at the respective doses [87]. Although usnic acid presents important biological activities, its low solubility is a limiting factor. Alternatively, its potassium salt has better solubility without compromising its biological potential [88]. The potassium salt usnic acid has been reported for promising schistosomicidal activity, causing mortality, motility changes, and tegument alterations in *Schistosoma mansoni*. Also, it has low toxicity to human cells, and therefore has high potential as a new anthelmintic drug for the control of schistosomiasis [89,90].

Numerous studies have investigated the potential of natural metabolites derived from algae and lichen for their anthelmintic activity against nematodes and schistosomiasis. However, there is limited research on their efficacy in acaricidal activity.

## 5. Acaricidal Activity of Microbial Metabolites

Many secondary metabolites produced by fungi and bacteria have been utilized in medicine and agriculture due to their diverse biological effects, including insecticidal, hypoglycemic, lipid-lowering, antitumor, anti-diabetic, antibacterial, and antifungal activities. *Beauveria bassiana* (*B. bassiana*) is a fungus that produces beauvericin (Figure 1-(**47**)), a secondary metabolite from the enniatin family [91]. *B. bassiana* toxins include various secondary metabolites and small molecular compounds, such as bassianin, beauvericin, bassianolide, tenellin, beauverolides, oxalic acid, oosporein, calcium oxalate crystals, and several beauvericin analogs [92]. Al Khoury et al. [22] assessed the potential acaricidal activity of beauvericin against various life stages of *S. scabiei*. They reported the first evidence of *B. bassiana*’s activity against *S. scabiei* eggs, with a hatching inhibition rate of 28.75%. Mechanistically, they found fungal genomic material within the surface-cleaned eggs that demonstrated the ability of *B. bassiana* to penetrate and proliferate within the eggshell of *S. scabiei* [93]. Chitinase (Figure 1-(**48**)) was induced in *Streptomyces mutabilis* IMA8 using chitin from *Charybdis smithii*, which exhibited potent miticidal activity (LC50, 24.2 ppm) against *D. gallinae* [94]. In vitro, *Metarhizium anisopliae* (*M. anisopliae*) CQMa128 demonstrated significant acaricidal activity against *P. cuniculi* in a time- and dose-dependent manner. Applying 6.14 × 10^9^ conidia/mL of *M. anisopliae* resulted in 83.33% mortality at 9 d, with an LT50 value of 6.1 d. In vivo, *M. anisopliae* achieved a 100% therapeutic effect after 3 d, compared to only 62.21% for ivermectin. The acaricidal activity of *M. anisopliae* was attributed to changes in enzyme activities within the detoxification and antioxidant system of *P. cuniculi* [95]. Emmanuel et al. [96] reported the in vitro acaricidal effect of *Bacillus thuringiensis* GP532 on *P. cuniculi* mites, with LC50 values of 1.3 mg/mL and 68 h. Furthermore, protein extracts from *B. thuringiensis* were shown to induce histological changes in *P. cuniculi*, including the enlargement of the basal membrane space, detachment of the peripheral nutrient matrix membrane, and morphological alterations in intestinal columnar cells.

## 6. Conclusions and Future Research Direction

Plant extracts, as one of the main sources of natural products, are eco-friendly and sustainable. They are readily available, biodegradable, and have high volatility, low-ecological toxicity, and low-environmental residual activity, with a huge development potential in controlling mites [97,98]. However, the current research mainly focuses on their in vitro acaricidal activity and only a few attempts have been made to their actual production. Meanwhile, the complex composition of these products limits the research on the acaricidal mechanism and it is difficult to establish a system standard for the extract [99]. The key mechanism of action of natural products is modification of the mite’s enzyme and interference with energy metabolism and nerve conduction (Figure 2). In the future, the application of multi-omics techniques, such as transcriptomics, proteomics, and metabolomics, can help to further explore the mechanism of mite killing by natural products at the molecular level [77,100].

Whether derived from plants, bacteria, or fungi, metabolite compositions are inherently complex. Though metabolomics is still in its developing stage, in the coming 10 years, there can be an impactful integration of LC-HRMS and NMR, allowing for the direct comparison and correlation of metabolite data [24]. While there are many studies on the biological activities of endophytic metabolites [101,102], such as antitumor [103,104,105], antibacterial [106], and insecticidal [107,108] activities, they lack in terms of acaricidal activities. This research area is believed to get the attention of scholars in the future.

Presently, it seems that natural products have limited anti-mite activity. However, structural modification and semi-synthesis of natural products can improve the anti-mite effect. Overall, natural products are promising molecular scaffolds for the development of new drugs, such as cinnamic acid [61,62,78,109], sanguinarine, chelerythrine [72,110], etc. Also, combining natural products with existing chemical drugs can reduce their resistance, decrease drug residue, and improve the therapeutic effect [111].

## Data Availability

Not applicable.
